# Surgical restoration of hand function in tetraplegia

**DOI:** 10.1038/s41394-021-00387-5

**Published:** 2021-03-19

**Authors:** Lina Bunketorp Käll, Johanna Wangdell, Carina Reinholdt

**Affiliations:** 1 Center for Advanced Reconstruction of Extremities C.A.R.E., Sahlgrenska University Hospital/Mölndal, Mölndal, Sweden; 2 Department of Health and Rehabilitation, Institute for neuroscience and physiology, Sahlgrenska Academy, The University of Gothenburg, Gothenburg, Sweden; 3 Department of Orthopaedics, Institute of Clinical Sciences, Sahlgrenska Academy, The University of Gothenburg, Gothenburg, Sweden

**Keywords:** Outcomes research, Spinal cord injury

## To the Editor:

It is with great interest that we read the illustrative case report by Hill et al. [1] in a recent issue of S*pinal Cord Series and Cases* (Hill EJ, El-Haj M, Giles JA, Fox IK. Using electrodiagnostics to define injury patterns amenable to nerve transfer surgery in tetraplegia: an illustrative case report. *Spinal Cord Series and Cases*. 2020; 6: 1–6.). In this report, the authors describe how patterns of neuronal injury due to cervical spinal cord injury (SCI) can be discerned by adding to the clinical examination electrodiagnostics to guide the timing and selection of nerve transfer to achieve optimal outcomes. The case concerned a 20-year-old man who had suffered a cervical SCI 8 months previously. Classified as American Spinal Cord Injury Association A at the C7 level, the injury caused dependency in activities of daily living. His goal was to regain active grip function so he could catheterize and feed himself.

Preoperative electrodiagnosis revealed a complex mixture of upper and lower motor neuron injuries in the intended recipient nerves originating from the C7–Th1 segments in the right upper limb. Specifically, the radial compound muscle action potential (CMAP) was absent and the ulnar CMAP was reduced, but the median CMAPs were intact, apart from absent activation of the flexor digitorum superficialis muscle. The absence of radial nerve CMAPs implied a mixture of upper and lower motor neuron injuries, whereas the intact median nerve indicated a functioning peripheral nerve and injured upper motor neurons. Intraoperative nerve stimulation confirmed a satisfactory motor response in the median nerve. Based on these results and the patient’s preferences and goals, the selection of nerve transfer procedures included transfer of the brachialis nerve to the anterior interosseous nerve and the flexor digitorum superficialis nerve fascicles of the median nerve to restore digit flexion and transfer of the supinator nerve to the posterior interosseous nerve to restore digit extension.

Although nerve transfers yield somewhat less predictable results than tendon transfers, we agree that selective nerve transfers offer exciting opportunities to restore function in tetraplegia [2]. They are more appealing than tendon transfer in some situations; for example, they allow direct reanimation of the muscle without altering its biomechanics and may produce finer motor control and more natural movements than tendon transfer. Further advantages include less extensive surgical dissection [3], shorter duration of hospitalization and rehabilitation, and fewer restrictions, thereby reducing health care use and cost [2]. Last but not least, selective nerve transfer is a fascinating alternative in the absence of locally suitable tendon transfer options, as in International Classification for Surgery of the Hand in Tetraplegia group 0 [4]. Another unique advantage is that sacrifice of a single nerve can potentially restore multiple paralyzed muscles. For example, the supinator motor nerves can be transferred directly to the posterior interosseous nerve to restore thumb and finger extension [5].

The main objective of the case report was to describe the use of electrodiagnostics to define injury patterns rather than to present the clinical outcome. Yet, we would like to comment on the surgical procedures and the functional outcome in this case. First, we would like to highlight the importance of selecting a restorative surgical procedure that does not risk loss of important donor muscle function. A well-chosen strategy such as harvesting brachialis nerve as in the present study, does not rule out the possibility of a salvage procedure, such as extensor carpi radialis brevis to flexor digitorum profundus tendon transfer, to reliably restore digit flexion. Harvesting the radial nerve branch to the extensor carpi radialis brevis as a donor to the anterior interosseous nerve would by contrast, risk losing important donor muscle function if the nerve reconstruction fail to produce a useful function. Second, we would like to highlight the importance of formulating a strategic plan for a second-stage procedure to salvage function when the primary nerve reconstruction does not produce the desired level of functional restoration. We support the view that hybrid reconstruction combining tendon transfer and nerve transfer may achieve greater potential gains than either technique in isolation [6].

For unstated reasons, the patient regrettably decided against additional tendon transfer surgery. Although he was said to accept an outcome with improved tenodesis grip by means of re-establishing volitional control of the flexor pollicis longus muscle, a second-stage tendon transfer would likely have produced a predictable active grasp, thereby better fulfilling his goal of regaining active grip function. The decision reflected the patient’s own preferences, which underscores the importance of involving the patient in decision-making through transparent discussions of the risks, uncertainties, potential benefits, and possible alternatives. Donating nerves for transfer has consequences should the intended reinnervation of peripheral denervated nerves prove unsuccessful, as it leaves the patient with no active finger flexion and continued dependency on the tenodesis grip as in the present case. The nerve transfers produced rather strong finger extension but a regrettably small increase in the strength of the flexor pollicis longus muscle (M2). Because a strong pinch or grasp is crucial to regain independence in activities of daily living, it does not greatly benefit patients functionally to restore only finger extensor function to enable active hand opening.

Tendon transfer is a powerful technique to restore independence and control in cases of tetraplegia, which is highly rewarding [2, 7–11]. In our experience, most patients believe the temporary inconvenience after a tendon transfer is well worth the predictable gains from the surgery. With support from previous studies [4, 6] and the cumulative experience from more than 1000 tendon transfer surgeries at the Swedish National Centre for Advanced Reconstruction of Extremities (Gothenburg, Sweden) and the Swiss Paraplegic Centre (Nottwil, Switzerland) [12], we would advocate a combined nerve and tendon transfer approach in a complex case like this. A two-stage procedure extends possibilities for reconstruction and potentially maximizes functional gains as compared to either technique alone, especially in patients with a complex mixture of upper and lower motor neuron injuries.

Exciting research is being done to advance knowledge in this fascinating field. Highly specialized comprehensive spinal units with upper extremity reconstruction facilities have an important part to play in this effort [12]. We agree with Drs. Hill and Fox that synchronized research is needed to compare nerve transfer with tendon transfer [13] and to find the optimal timing of those procedures, whether done alone or, more importantly, in combination.

## References

[1] Hill EJ, El-Haj M, Giles JA, Fox IK (2020). Using electrodiagnostics to define injury patterns amenable to nerve transfer surgery in tetraplegia: an illustrative case report. Spinal Cord Ser Cases.

[2] Fridén J, Gohritz A (2015). Tetraplegia management update. J Hand Surg Am.

[3] O’grady KM, Power HA, Olson JL, Morhart MJ, Harrop AR, Watt MJ (2017). Comparing the efficacy of triple nerve transfers with nerve graft reconstruction in upper trunk obstetric brachial plexus injury. Plast Reconstr Surg.

[4] Brown JM. Nerve transfers in tetraplegia I: background and technique. Surg Neurol Int. 2011;2:121. 10.4103/2152-7806.84392 PMC3172032 21918736

[5] Bertelli JA, Ghizoni MF (2015). Nerve transfers for elbow and finger extension reconstruction in midcervical spinal cord injuries. J Neurosurg.

[6] Cavallaro DC, Mikalef P, Power DM (2019). A comparison of tendon and nerve transfer surgery for reconstruction of upper limb paralysis. J Musculoskel Surg Res.

[7] Bunketorp-Kall L, Wangdell J, Reinholdt C, Friden J (2017). Satisfaction with upper limb reconstructive surgery in individuals with tetraplegia: the development and reliability of a Swedish self-reported satisfaction questionnaire. Spinal Cord.

[8] Wangdell J, Carlsson G, Friden J (2014). From regained function to daily use: experiences of surgical reconstruction of grip in people with tetraplegia. Disabil Rehabil.

[9] Wangdell J, Carlsson G, Fridén J (2013). Enhanced independence: experiences after regaining grip function in people with tetraplegia. Disabil Rehabil.

[10] Wangdell J, Friden J (2010). Satisfaction and performance in patient selected goals after grip reconstruction in tetraplegia. J Hand Surg Eur Vol.

[11] Bunketorp-Käll L, Reinholdt C, Fridén J,Wangdell J. Essential gains and health after upper-limb tetraplegia surgery identified by the International classification of functioning, disability and health (ICF). Spinal Cord. 2017; 10.1038/sc.2017.36. 10.1038/sc.2017.36 28418396

[12] Fridén J, Lieber RL. Reach out and grasp the opportunity: reconstructive hand surgery in tetraplegia. J Hand Surg. 2019;44:343–53. 10.1177/1753193419827814 PMC9075705 30744461

[13] Hill EJ, Fox IK (2019). Nerve transfers to restore upper limb function in tetraplegia. Lancet..

